# Corneal transplantation outcomes after the extrusion of an intrastromal keratoprosthesis: a pilot study

**DOI:** 10.1186/s40662-020-00193-4

**Published:** 2020-05-08

**Authors:** Chiara Fariselli, Ibrahim Toprak, Olena Al-Shymali, Jorge L. Alio del Barrio, Jorge L. Alio

**Affiliations:** 1 Research and Development Department, Calle Cabañal, 1 Edificio Vissum, 03016 Alicante, Spain; 2 Department of Ophthalmology, Pamukkale University, Denizli, Turkey; 3 Cornea and Refractive Surgery Department, Calle Cabañal, 1 Edificio Vissum, 03016 Alicante, Spain; 4 Division of Ophthalmology, Universidad Miguel Hernández, Alicante, Spain

**Keywords:** Keratoprosthesis, Extrusion, Keratoplasty, Corneal transplantation

## Abstract

This short report includes 5 eyes of 5 patients (mean age 63.2 ± 12 years) who underwent a tectonic keratoplasty [deep anterior lamellar keratoplasty (DALK) or penetrating keratoplasty (PK)] in order to rehabilitate the eye after the extrusion of the non-perforating keratoprosthesis (Kpro) KeraKlear (KeraMed, USA). The non-perforating Kpro was extruded after a mean period of 21.4 ± 21.8 months due to melting. In two cases, the keratoplasty was performed the same day of the non-perforating Kpro removal due to a severe melting, while in the other three cases it was performed one to 3 months later. Two eyes received a DALK, but in 3 eyes a macroscopic Descemet membrane perforation forced the conversion into a PK. The mean follow-up period after the keratoplasty was 16.8 ± 6.6 months. No cases of rejection were recorded. All the 5 eyes achieved “anatomical success” (transparent graft, with no signs of infection or inflammation). Two eyes showed limited “functional success” because the achievement of the best visual potential was prevented by the development of glaucomatous optic atrophy during the follow-up period. In conclusion, this short report presents an unexpected success of a keratoplasty performed with a tectonic purpose after the extrusion of the non-perforating Kpro because the corneal graft remained transparent, without neovascularization or scarring during the follow-up period. This initial evidence shows some encouraging results regarding graft survival rate and the achievement of a useful visual rehabilitation with keratoplasty after a non-perforating Kpro failure instead of repeating the Kpro implantation.

## Background

Keratoprostheses (Kpros) represent a useful surgical option for patients with corneal blindness affected by conditions at high risk of failure with standard penetrating keratoplasty (PK) [1–4]. However, the retention failure is a notable problem for this type of surgery [5–7]: a sterile keratolysis, in fact, can result in corneal perforation, leading to extrusion of the device and final Kpro failure [8]. Eyes that fail initial Kpro implantation due to melting may either be rehabilitated undergoing repeat Kpro implantation or a tectonic keratoplasty [9]. A particular type of Kpro is the KeraKlear Kpro (KeraMed, USA), which is made of biocompatible acrylic material, with peripheral holes that facilitate fixation, hydration and nutrition of the remaining cornea. The KeraKlear Kpro implantation requires a non-perforating surgery therefore the postoperative risk of intraocular infection as well as the risks of expulsive hemorrhage and glaucoma worsening are reduced [10]. In this short report, we present the outcomes of keratoplasty performed after the extrusion of the KeraKlear Kpro.

## Methods

This pilot study short report is about the feasibility of corneal transplantation following anatomical failure of intrastromal keratoprosthesis and includes 5 eyes of 5 patients who underwent a penetrating keratoplasty (PK) or a deep anterior lamellar keratoplasty (DALK) after the extrusion of a previously implanted KeraKlear Kpro. The procedure of the investigation conformed to the tenets of the Declaration of Helsinki. All patients provided written consent and institutional review board approval was obtained from Vissum Alicante. The non-perforating corneal prosthesis KeraKlear had been implanted in order to treat a corneal blindness caused by conditions at high risk of failure with standard penetrating keratoplasty: multiple failed previous PK in one eye and extensive limbal stem cell deficiency with corneal vascularization and scarring in 4 eyes. In all cases, the neovascularization involved the four quadrants and, depending on the case, a total or partial stromal scarring and opacification was present. During the selection for Kpro implantation, patients were not excluded based on the visual acuity of the fellow eye. The KeraKlear Kpro doesn’t need a total corneal trephination, because it is a non-perforating Kpro, which is inserted into the cornea: onto the deep stroma (intralamellar technique) or over the Descemet’s membrane (DM) (epidescemetic technique). In all the five eyes, the implantation was performed assisted by femtosecond laser (IntraLase 60 kHz; Abbott Medical Optics, Santa Ana, California, USA). An 8 mm corneal diameter dissection was done at 100 μm from the DM and a second corneal dissection of 3.5 mm was done at a shallower depth. The central 3.5 mm disc of the anterior cornea was removed and then dissected manually by a crescent knife. In the epidescemetic technique, the corneal stroma was removed by manual dissection or assisted by a big bubble. The KeraKlear Kpro was put directly onto the DM and the corneal graft was then sutured to the recipient as for DALK [10]. Avoiding the invasion of the anterior chamber, the risk of intraocular infection, expulsive hemorrhage, worsening glaucoma and necrosis of tissue around the device are reduced, when compared with Boston Kpro [10]. During the follow-up period after the non-perforating Kpro implantation, all 5 eyes developed an untreatable melting. Systemic doxycycline, topical platelet-rich plasma and temporary use of bandage contact lens were tried in order to contrast the sterile keratolysis, but the severe corneal melting led to the extrusion of the Kpro. Furthermore, when the tissue necrosis was so advanced to be untreatable, the Kpro could not be left in place due to the risk of intraocular infection and loss of the eye. Thus, a tectonic keratoplasty was performed (DALK in 2 eyes and PK in 3 eyes).

The time between the KeraKlear Kpro extrusion and the corneal transplantation was evaluated, and it was different according to condition of the eye. If the eye presented a really severe melting, involving more than a half of the cornea, with a high risk of perforation and loss of the eye, the tectonic keratoplasty was performed the same day of the Kpro removal. If the KeraKlear Kpro was extruded, but the eye was quiet, without any risk of perforation, the time necessary for corneal healing was allowed to pass and the keratoplasty was performed one to 3 months later. In this second scenario, between the KeraKlear Kpro extrusion and the keratoplasty, a bandage contact lens was applied and patients were reviewed every week, with careful slit lamp examination and anterior segment optical coherence tomography (AS-OCT, Visante OCT, Carl Zeiss, Germany). The preparation of the donor cornea was performed by the femtosecond laser in three cases out of five; in the other two cases the cut was performed manually. In all cases, a lamellar keratoplasty (DALK) was tried initially, and if a macroscopic DM and endothelium perforation occurred during the surgery, surgical procedure was converted into a full thickness PK. All procedures were performed by the same expert surgeon (JA). After the keratoplasty, all patients underwent a careful visit the first postoperative day, one, two and 4 weeks after the surgery, and then every 3 months. The best corrected visual acuity was recorded. All grafts were classified at a “very high risk” of failure and were treated with the following therapy: fluoroquinolone drops every 6 h for 1 week (or until complete epithelial closure); dexamethasone 0.1% drops every 2 h during the day, tapering the frequency according to clinical response, keeping at least once a day indefinitely; tacrolimus 0.03% ointment at night, for 6 months to 1 year; oral prednisolone 10 mg once a day for 1 month, then 5 mg once a day for another 2 months; oral tacrolimus 1 mg every 12 h for 1 year; mycophenolate mofetil 500 mg every 12 h for 1 year, and reduced to 250 mg, prior to evaluation of the tolerance. The time of follow-up after the keratoplasty and the rate of anatomical/functional success were studied. The “anatomical success” was defined by a transparent graft, with no signs of infection or inflammation, independently of the visual performance, that was very limited in some cases due to ocular comorbidities. The “functional success” was defined by the achievement of the best visual potential which was expected considering the graft transparency.

## Results

Five eyes of five patients (mean age 63.2 ± 12 years) underwent the implantation of the KeraKlear Kpro because of conditions at high risk of failure for standard keratoplasty. After a mean period of 21.4 ± 21.8 months, melting led to the extrusion of the Kpro, and in order to rehabilitate the eye, a tectonic keratoplasty was performed. In two cases, the keratoplasty was performed the same day of the KeraKlear Kpro removal, while in the other three cases it was performed 1, 2 and 3 months later. The mean period between the KeraKlear Kpro extrusion and the keratoplasty was of 1.4 ± 1.1 months. Patients outcomes are summarized in Table 1.

**Table 1:** Outcomes of the 5 eyes that underwent keratoplasty after the extrusion of the non-perforating KeraKlear keratoprosthesis

Patient	Age (years)	Ocular diagnosis (first diagnosis - when available)	Pre-Kpro VA (Snellen equivalent)	Post-Kpro VA (Snellen equivalent)	Time between Kpro implantation and Kpro extrusion (months)	Time between Kpro extrusion and keratoplasty (months)	Keratoplasty [diameter]	Post-keratoplasty VA (Snellen equivalent)	Anatomical success [cause; final VA] or functional success	Follow-up after the keratoplasty (months)
1	67	3 previous failed PK (PBK)	CF at 10 cm	CF at 1 m	41	0	PK [donor 9.5 mm, recipient 9 mm]	0.16	Anatomical success [glaucomatous optic nerve atrophy; CF at 2 m]	24
2	72	LSCD	LP	CF at 50 cm	48	3	PK [donor 8 mm, recipient 7.75 mm]	0.05	Anatomical success [glaucomatous optic nerve atrophy; 0.05]	12
3	60	LSCD (pemphigoid)	LP	0.16	14	0	DALK [donor 8 mm, recipient 7.75 mm]	0.05	Functional success	12
4	73	LSCD	0.05	0.067	3	2	PK [donor 8 mm, recipient 7.75 mm]	0.68	Functional success	24
5	44	LSCD (necrotizing herpetic stromal keratitis)	HM	CF at 10 cm	1	1	DALK [donor 8.3 mm, recipient 8 mm]	0.05	Functional success	12

*PBK* = pseudophakic bullous keratopathy; *CF* = counting fingers; *DALK* = deep anterior lamellar keratoplasty; *Kpro* = keratoprosthesis; *LSCD* = limbal stem cell deficiency; *PK* = penetrating keratoplasty; *LP* = light perception; *VA* = visual acuity (decimal scale); *HM* = hand motion

In all cases a lamellar keratoplasty was tried initially, but in three eyes a macroscopic DM perforation occurred and the surgical procedure was converted into a PK. Thus, 2 eyes received DALK and 3 eyes received PK. All eyes received 16 interrupted 10–0 nylon sutures, except one case that received a double running suture as per the surgeon’s criteria. The sutures were removed 1 year later for the PKs and 8 to 9 months after the DALKs. The mean follow-up period after the keratoplasty was 16.8 ± 6.6 months.

**Fig. 1:**
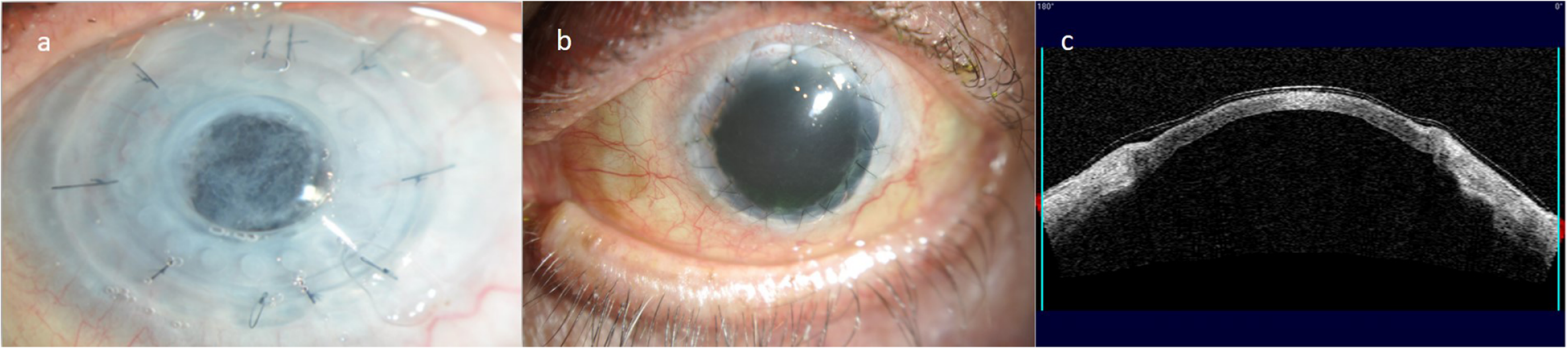
Clinical presentation of Patient 1, before the extrusion of KeraKlear Kpro and after the keratoplasty. a) Non-perforating Keratoprosthesis (Kpro), b) Transparent corneal graft after Penetrating Keratoplasty (PK), c) AS-OCT of the PK

**Fig. 2:**
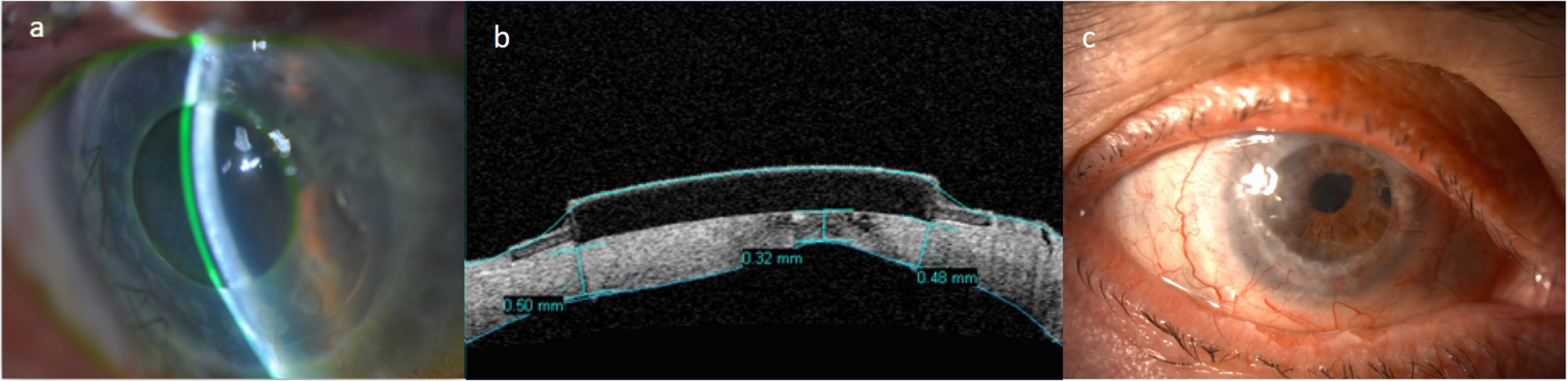
Clinical presentation of the extrusion of KeraKlear Kpro in Patient 4 that led to keratoplasty. a) Non-perforating Keratoprosthesis (Kpro), b) AS-OCT of the extrusion of the Kpro, c) Transparent corneal graft

Patient 1 (Fig. 1), during the follow-up period after keratoplasty, developed an increase in intraocular pressure, which required the implantation of an ex-press valve 18 months after the PK. In spite of the glaucomatous surgery, the final visual acuity was compromised by the damage to the optic nerve. Patient 2 suffered from glaucoma, and after the PK presented a worsening of the glaucomatous disease along with development of an optic nerve atrophy. During the follow up after the keratoplasty, two eyes developed a corneal ulcer. The first case was patient 3 who was treated with bandage contact lens, platelet rich plasma and antibiotics. In patient 4 (Fig. 2), during the development of the ulcer, a herpetic disease was suspected. The polymerase chain reaction was positive for herpes simplex, so topical and systemic acyclovir were able to control and heal the keratitis.

During the follow-up period, no eye was lost and all the 5 eyes achieved anatomical success: a transparent graft, with no graft neovascularization and no inflammation (no cases of rejection were recorded). Two eyes showed limited functional success due to the development of glaucomatous optic atrophy during the follow-up period.

## Discussion

A significant problem for Kpro implantation, including also the KeraKlear, is the retention failure. In our case series, the extrusion of the implant occurred after an average period of 21.4 ± 21.8 months, and a keratoplasty with tectonic purpose had to be performed. Several new techniques have been reported in order to manage the Kpro failure caused by sterile corneal stromal melts, such as crescentic amniotic membrane grafting and buccal mucosal membrane graft, but no data about the long-term efficacy of these techniques are available [11–13]. Repeat Kpro implantation and tectonic keratoplasty are the two more feasible techniques for the management of failed Kpro. The comparison between repeat Kpro implantation and tectonic keratoplasty after Boston Kpro failure showed favorable long-term visual outcomes in patients undergoing repeat Kpro implantation [9, 14]. However, in our case series, repeating the non-perforating Kpro implantation was not indicated due to the lack of adequate clinical conditions. Therefore, a keratoplasty was performed after the failure of the non-perforating Kpro, just with a tectonic purpose. But during the follow-up period, we noticed an unexpected success of the keratoplasty. The corneal graft remained transparent without graft neovascularization or scarring, and thus allowing the patient to achieve a useful visual rehabilitation.

At the end of XIX^th^ century, a military surgeon, Johann Friedrich August Von Esmarch (1823–1908), noted that the elimination of local infection, necrotic tissue or malignancy prevents diseased tissue to affect the adjacent areas, with improvement in the clinical condition of the residual tissue [15]. From this evidence, raised in a period in which no antibiotics, corticosteroids or anti-inflammatory medications were available, led to the general acceptance of this technique (“the von Esmarch technique”) in order to improve the outcome of severe wounds. We may speculate that in our cases, excision of the diseased cornea required for the Kpro intrastromal implantation might have induced regression of the neovessels and the scars in the remaining tissue. Surprisingly, corneas that initially were not able to bear a keratoplasty due to the extent of neovascularization and limbal stem cell deficiency, after the extrusion of the non-perforating Kpro and the performance of a tectonic keratoplasty had showed the ability to host the new tectonic corneal graft successfully, allowing also for some visual rehabilitation.

## Conclusions

KeraKlear Kpro can provide, in some eyes, a temporary solution to corneal blindness for those cases not approachable with regular corneal graft transplantation techniques. However, in the event of extrusion, the initial evidence provided in this paper shows some encouraging results regarding the short-term visual outcomes and graft survival rate of a repeat corneal graft instead of redo-Kpro. Close follow-up to control postoperative complications and systemic immunosuppression is essential for these patients.

## Data Availability

The datasets used and/or analyzed during the current study are available from the corresponding author on reasonable request.
